# Inverse treatment planning for spinal robotic radiosurgery: an international multi‐institutional benchmark trial

**DOI:** 10.1120/jacmp.v17i3.6151

**Published:** 2016-05-08

**Authors:** Oliver Blanck, Lei Wang, Wolfgang Baus, Jimm Grimm, Thomas Lacornerie, Joakim Nilsson, Sergii Luchkovskyi, Isabel Palazon Cano, Zhenyu Shou, Myriam Ayadi, Harald Treuer, Romain Viard, Frank‐Andre Siebert, Mark K.H. Chan, Guido Hildebrandt, Jürgen Dunst, Detlef Imhoff, Stefan Wurster, Robert Wolff, Pantaleo Romanelli, Eric Lartigau, Robert Semrau, Scott G. Soltys, Achim Schweikard

**Affiliations:** ^1^ Department of Radiation Oncology University Medical Center Schleswig‐Holstein Kiel Germany; ^2^ Department of Radiation Oncology Saphir Radiosurgery Center, Frankfurt am Main & Güstrow Germany; ^3^ Department of Radiation Oncology Stanford University Stanford CA USA; ^4^ Department of Radiation Oncology University Hospital Cologne Cologne Germany; ^5^ Bott Cancer Center, Holy Redeemer Hospital Meadowbrook PA USA; ^6^ Academic Radiation Oncology Department CLCC Oscar Lambret, & University Lille II Lille France; ^7^ Department of Radiation Oncology CyberClinic Cancer Center Spizhenko Kyiv Ukraine; ^8^ Department of Medical Physics Hospital Ruber Internacional Madrid Spain; ^9^ Department of Radiation Oncology CyberKnife Center Tampa Bay Tampa Bay FL USA; ^10^ Radiation Therapy Department Centre Léon Bérard Lyon France; ^11^ Department of Stereotaxy and Functional Neurosurgery University Hospital Cologne Cologne Germany; ^12^ AQUILAB Loos les Lille France; ^13^ Department of Radiation Oncology Tuen Mun Hospital Hong Kong Hong Kong; ^14^ Department of Radiation Oncology University Hospital Rostock Rostock Germany; ^15^ Department of Radiation Oncology University Hospital Copenhagen Copenhagen Denmark; ^16^ Department of Radiation Oncology University Hospital Frankfurt Frankfurt am Main Germany; ^17^ Brain Radiosurgery & CyberKnife Center, Centro Diagnostico Italiano Milano Italy; ^18^ Institute for Robotic and Cognitive Systems, University of Lübeck Lübeck Germany

**Keywords:** CyberKnife robotic radiosurgery, benchmark study, inverse treatment planning, optimization

## Abstract

Stereotactic radiosurgery (SRS) is the accurate, conformal delivery of high‐dose radiation to well‐defined targets while minimizing normal structure doses via steep dose gradients. While inverse treatment planning (ITP) with computerized optimization algorithms are routine, many aspects of the planning process remain user‐dependent. We performed an international, multi‐institutional benchmark trial to study planning variability and to analyze preferable ITP practice for spinal robotic radiosurgery. 10 SRS treatment plans were generated for a complex‐shaped spinal metastasis with 21 Gy in 3 fractions and tight constraints for spinal cord ($V_{14\text{Gy}}<2\;\text{cc}$, $V_{18\text{Gy}}<0.1\;\text{cc}$) and target (coverage >95%). The resulting plans were rated on a scale from 1 to 4 (excellent‐poor) in five categories (constraint compliance, optimization goals, low‐dose regions, ITP complexity, and clinical acceptability) by a blinded review panel. Additionally, the plans were mathematically rated based on plan indices (critical structure and target doses, conformity, monitor units, normal tissue complication probability, and treatment time) and compared to the human rankings. The treatment plans and the reviewers' rankings varied substantially among the participating centers. The average mean overall rank was 2.4 (1.2‐4.0) and 8/10 plans were rated excellent in at least one category by at least one reviewer. The mathematical rankings agreed with the mean overall human rankings in 9/10 cases pointing toward the possibility for sole mathematical plan quality comparison. The final rankings revealed that a plan with a well‐balanced trade‐off among all planning objectives was preferred for treatment by most participants, reviewers, and the mathematical ranking system. Furthermore, this plan was generated with simple planning techniques. Our multi‐institutional planning study found wide variability in ITP approaches for spinal robotic radiosurgery. The participants', reviewers', and mathematical match on preferable treatment plans and ITP techniques indicate that agreement on treatment planning and plan quality can be reached for spinal robotic radiosurgery.

PACS number(s): 87.55.de

## I. INTRODUCTION

Hallmarks of stereotactic radiosurgery (SRS) and stereotactic body radiation therapy (SBRT) include the accurate, conformal delivery of high‐dose radiation to targets while minimizing normal tissue irradiation via precise target localization^(1)^ and steep dose gradients through multiple beam directions.^2^, ^3^ Most SRS/SBRT delivery systems today incorporate complex target localization and motion compensation strategies^4^, ^5^ and allow for nearly limitless possibilities for beam shapes, orientations, motion, and intensities, making it cumbersome, if not impossible, to create forward‐planned treatment plans in routine practice. Therefore, almost all treatment planning systems use inverse treatment planning (ITP) with a variety of optimization algorithms.^6^, ^7^, ^8^, ^9^, ^10^ Recently, multicriteria optimization^11^, ^12^ to accommodate different clinical preferences and conflicting optimization objectives (e.g., maximizing tumor coverage while minimizing normal tissue doses) have been added to the increasing complexity of computer‐aided treatment planning.

Yet, not all possible beam configurations can be simulated, due to computational and temporal constraints. Therefore, the quality of treatment planning remains user‐dependent as manual preselection of optimization and beam parameters are generally required.^13^, ^14^ Additionally, the background training and experience of the treatment planner can vary significantly. A general quality measure for the treatment planning process or the treatment planner itself does not exist and a best practice guideline is largely missing for all radiotherapy systems in clinical practice. To make a first step to overcome this shortage, we performed an international, multi‐institutional treatment planning benchmark trial to analyze treatment planning variability and to analyze best practice for treatment planning for spinal robotic radiosurgery with the CyberKnife (Accuray Incorporated, Sunnyvale, CA).^(15)^

## II. MATERIALS AND METHODS

### A. Treatment planning for robotic radiosurgery

Treatment planning for CyberKnife with the MultiPlan Treatment Planning System (version 4.5) (Accuray) is based on inverse planning using linear optimization.^(9)^ In the first step, 500‐1500 beams per manually selected cylindrical beam size (5‐60 mm) are randomly oriented toward target surface points from approximately 120‐180 precalibrated linear accelerator positions around the patient couch, resulting in 1500‐6000 noncoplanar nonisocentric beams. Alternatively, a strictly isocentric beam arrangement can be generated, which is rarely used for complex‐shaped targets as it limits the flexibility during optimization. In the second step, a large inequality matrix is generated based on the calculated beam dose coefficients of each voxel of the discretized contours and manually predefined dose constraints. A weighted cost function representing the planning objectives is then minimized, using linear programming (LPSolve, SourceForge or CPLEX, IBM ILOG), to determine the optimal monitor unit (MU) for each beam, which generally results in 50‐350 treatment beams, depending on case complexity and beam size selection.^(15)^

Previously the whole cost function was minimized simultaneously using manually set weights for the individual objectives (MultiPlan versions prior to version 3.0). Sequential Multi‐Objective Optimization (SMOO; introduced in 2008 with MultiPlan version 3.0) minimizes each term of the cost function in a stepwise procedure in which the result of each step becomes a constraint in the next step subtracted by a manually set relaxation value.^(11)^ Therefore, SMOO facilitates the exploration of maximum trade‐offs between different objectives like maximal target coverage, minimal critical structure dose, maximal dose conformity using shell structures or minimal total monitor units. Dose‐volume optimization,^(16)^ (pseudo) dose‐volume‐constraints, resampling,^(17)^ and reoptimization with removed low MU beams further added to the flexibility during this interactive treatment planning process. Initial studies with SMOO and multiple collimators demonstrated that superior plan quality was achievable over simultaneous optimization,^(18)^ but SMOO also introduced much more user‐dependent variability in planning, with possible resultant variability in treatment plan quality.

### B. Treatment planning benchmark

**Figure 1:**
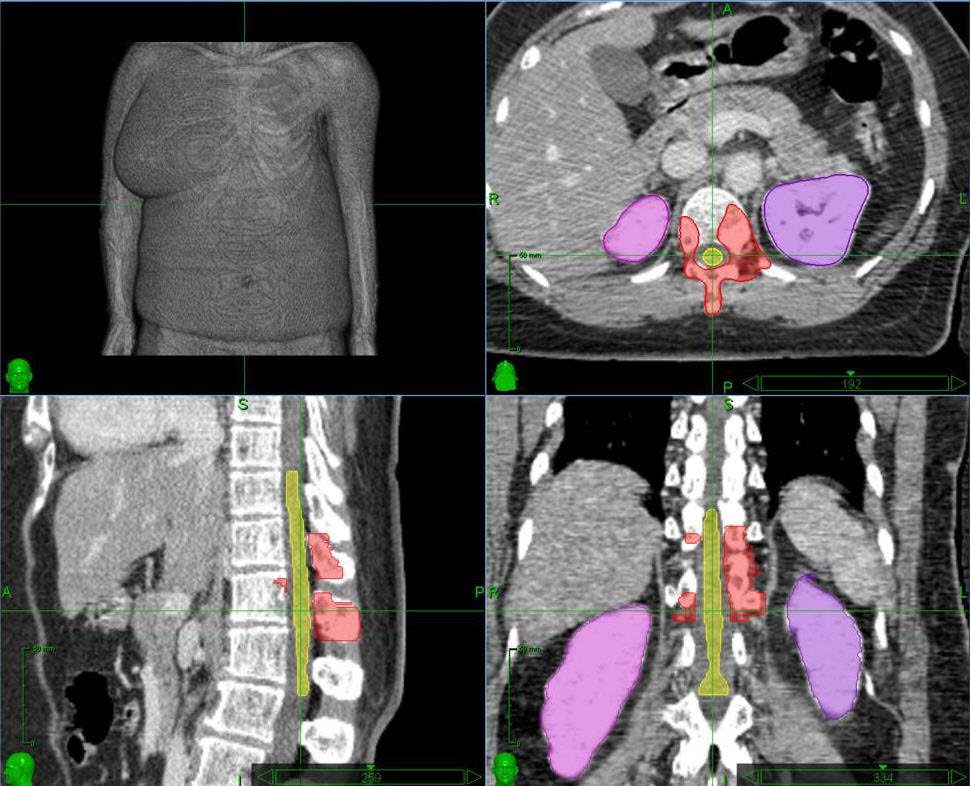
Axial, sagittal, and coronal view of the planning target volume (red), spinal cord (yellow), and kidneys (purple) for the spinal benchmark case.

A single, complex‐shaped, recurrent, previously irradiated spinal metastasis (see Fig. 1) was selected for this study. The planning target volume (PTV) was 40.2 cc (maximum dimension 4.7×5.6×6.1 cm, median beam's eye view segment size approx. 2 cm), located in the lower thoracic region (T11/T12) and close to the kidneys. All volumes of interest were defined on CT/MR fusion images. Given the prior fractionated irradiation to the spinal cord, a conservative target dose of 21 Gy in 3 fractions with strict limits for the spinal cord ($V_{14\text{Gy}}<2\;\text{cc}$, $V_{18\text{Gy}}<0.1\;\text{cc}$) was chosen. Higher target doses of 27‐30 Gy in 3 fractions may have been led to higher local control;^19^, ^20^ however, the given limitations for the spinal cord due to multiple preirradiations did not allow a higher dose for this patient. A secondary defined constraint was the dose to the kidney ($D_{\text{max}}<22\;\text{Gy}$).

The planning objectives were a) not to exceed the spinal cord limits and to achieve at least 95% of the prescribed dose (21 Gy) covering the PTV, and b) to maximize conformity of the prescribed isodose to the PTV (due to previous irradiation and risk of fracture outside the target area) and to minimize total monitor units and treatment time alike (due to back pain of the patient). Objectives not explicitly mentioned to the participants were: to optimize PTV minimum dose, spinal cord, kidney, and skin maximum doses and dose to the healthy tissue surrounding the PTV. Ten dedicated medical physicists or dosimetrists from eight CyberKnife centers volunteered to generate a clinically acceptable treatment plan based on the above planning objectives according to their standard code of practice. Treatment planning was performed on a single, dedicated, remotely accessible MultiPlan station within a 24‐hr timeslot to simulate realistic clinical practice. All participants were blinded to other participants' treatment plans, which was ensured by anonymization and computer log file monitoring during the study.

### C. Review panel ranking

All 10 anonymous cases were then reviewed by an independent review panel of three neurosurgeons, five radiation oncologists, and two medical physicists who did not participate in the study. Each review panel member rated the 10 cases based on a scale from 1 to 4, with 1 being good or excellent, 2 being average, 3 being below average, and 4 being poor for a) meeting the main clinical objectives (spinal cord $V_{14\text{Gy}}<2\;\text{cc}$ and $V_{18\text{Gy}}<0.1\;\text{cc}$, 95% PTV coverage), b) optimizing secondary objectives (conformity, monitor units, treatment time), and c) optimizing objectives not explicitly mentioned (PTV $D_{\text{min}}$, spinal cord $D_{\text{max}}$, kidney $D_{\text{max}}$, skin $D_{\text{max}}$, dose gradient). The physicists also rated d) the complexity of the ITP process (tuning structures, collimator selection, constraint selection, shell structures, optimization script), and the clinicians rated e) the clinical acceptability of the treatment plan (treatment complexity, potential treatment flaws, dose distribution, trade‐offs between conflicting objectives). Each final plan ranking was calculated based on the sum of the ratings of the individual categories (A‐E) and a normal distribution (bell curve) of the scale mentioned above (1‐4) over the lowest and highest sum.

### D. Mathematical ranking

In addition to the empirical ranking by the review panel, we also performed a mathematical ranking based on plan indices to explore potential automated plan quality comparison. We used the secondary plan review software ARTIVIEW (AQUILAB, Loos les Lille, France) to calculate all plan indices based on the original plans. On the basis of the planning objectives and the review panel ranking, the following indices were analyzed in three categories: A) spinal cord $V_{14\text{Gy}}$ and $V_{18\text{Gy}}$ and PTV coverage at 21 Gy (prescription dose), B) conformity index (ratio between the total volume receiving 21 Gy and the PTV), total monitor units, and the estimated treatment time (includes patient setup, robot motion, and beam‐on time), and C) PTV $D_{\text{min}}$, spinal cord $D_{\text{max}}$, kidney $D_{\text{max}}$, 5 mm skin $D_{\text{max}}$, and volume10 cm $V_{10\text{Gy}}$ (the volume receiving 10 Gy in the 10 cm volume surrounding the PTV).

Additionally, for Category A, we calculated a potential represcription dose for the PTV based on the normal tissue complication probability (NTCP) for the spinal cord. To estimate the spinal cord myelopathy NTCP, we used the DVH Evaluator (DiversiLabs, Biomérieux, Durham, NC) and published literature for spinal cord tolerances^21^, ^22^ resulting in spinal cord limits of $V_{18\text{Gy}}<0.1\;\text{cc}$ and $V_{16\text{Gy}}<1\;\text{cc}$. We then represcribed the dose so that the spinal cord $V_{18\text{Gy}}$ was lower than or equal to 0.1 cc and the spinal cord $V_{16\text{Gy}}$ was lower than or equal to 1 cc and noted the PTV $D_{95\%}$, the dose received by 95% of the PTV. Without this step, each plan has a different PTV coverage dose and a different NTCP risk level, making comparisons more complex. Therefore we essentially hold NTCP fixed for all cases, and represcribed each plan to the corresponding highest possible $D_{95\%}$ to compare tumor dose more clearly. Finally, we rated each index based on the same scale (1‐4) mentioned above, using the normal distribution (bell curve) of the best and the worst results for that index. The final plan ranking was also calculated as above.

## III. RESULTS

### A. Treatment planning approaches

The treatment planning approaches varied substantially among various participating CyberKnife centers and their designated treatment planner (TP) for this study (Table 1). The optimization script also varied substantially among the TP ranging from 1 to 17 SMOO steps (median 5 steps). Six TP (60%) used dose‐volume constraints and only five TP explicitly constrained the kidney maximum dose. Three TP optimized minimum PTV dose, while the others optimized PTV coverage in the first step, with only one TP using dose‐volume optimization. After PTV optimization, six TP (60%) focused on spinal cord optimization, while two (20%) focused on dose falloff by reducing the shell structures' maximum doses. Five TP (50%) optimized at the end the monitor units which did not adhere to the initial maximum MU limits. Only one TP (10%) used only one optimization step and manually adjusted critical organs, tuning shells, and MU constraints in multiple reoptimization iterations (TP case 10). For beam and monitor unit (MU) constraints, four TP (40%) explicitly limited the maximum MU, while three TP (30%) limited the MU through collimator selection, and three TP (30%) relied solely on artificial shell structures to limit the skin and hot spot doses. Collimator sizes between 10‐25 mm were generally preferred for this treatment agreeing with the median beam's eye view PTV segment size, with overall median collimator sizes per TP of 20 mm (min 12.5 mm, max 40 mm). The Iris (Accuray Inc.) variable aperture collimator was used by five TP (50%), while the other five noted that they did not use the Iris collimator clinically.

**Table 1:** Treatment approaches of the different participating centers.

	*Case*								
	*C 1*	*C 2*	*C 3*	*C 4*	*C 5*	*C 6*	*C 7*	*C 8*	*C 9*	*C 10*
*Plan Setup*										
Beam Block	n/a	n/a	Arms	Arms	n/a	n/a	n/a	n/a	n/a	n/a
Max MU/Node	n/a	600	750	850	1400	550	900	n/a	900	925
Max MU/Beam	600	300	735	500	450	290	400	300	450	425
Max Total MU	36000	40000	n/a	n/a	n/a	n/a	n/a	n/a	45000	41000
Shell Structures	4	3	8	4	3	2	2	2	3	4
Tuning Structures	n/a	n/a	n/a	2	n/a	1	n/a	1	n/a	n/a
Collimator Iris/Fixed	Iris	Iris	Fixed	Fixed	Iris	Fixed	Fixed	Fixed	Iris	Iris
(sizes in mm)	7.5‐25	12.5‐30/40	7.5‐25/40	10/15/20	7.5/10/15‐25	12.5/25	7.5/15/30	25/40/60	7.5‐20	10/15/20/35
Median Coll. Size (mm)	15.0	25.0	20.0	15.0	15.0	20.0	20.0	40.0	12.5	20.0
*Optimization*										
Spinal Cord Max (cGy)	1820	1600	1668	1750	1750	1750	1740	2000	1800	1850
Volume Constraint (cc)	n/a	n/a	n/a	V14<1.7	n/a	V14<2	V14<1.8	V18<0.1	V14<2	V14.8<2
Kidney Constraints	N	Y	Y	N	N	Y	N	N	Y	Y
OCO/OMI /DVL	OCO	OCO	OMI/CO	OMI/CO	OCO	OCO	OCO	OMI/CO	OCO	DVL
OMA/OME/DVU	OME	DVU	OMA	OME	n/a	n/a	OMA	n/a	OMA	n/a
OME Kidney Y/N	N	Y	Y	Y	N	N	N	N	N	N
Shell Structures	2	0	8	2 Tuning	3	1	2	0	3	0
OMU Y/N	N	Y	Y	N	Y	Y	Y	N	N	N
Optimization Steps	4	5	17	8	5	3	5	2	5	1

OCO = optimize coverage; OMI/CO = Optimize Min Dose first and second Optimize Coverage; DVL = dose volume optimization; OMA = optimize max dose; OME = optimize mean dose; DVU = dose‐volume optimization; OMU = optimize monitor units; Y = yes; N = no; n/a = not assigned.

### B. Resulting treatment plans

**Figure 2:**
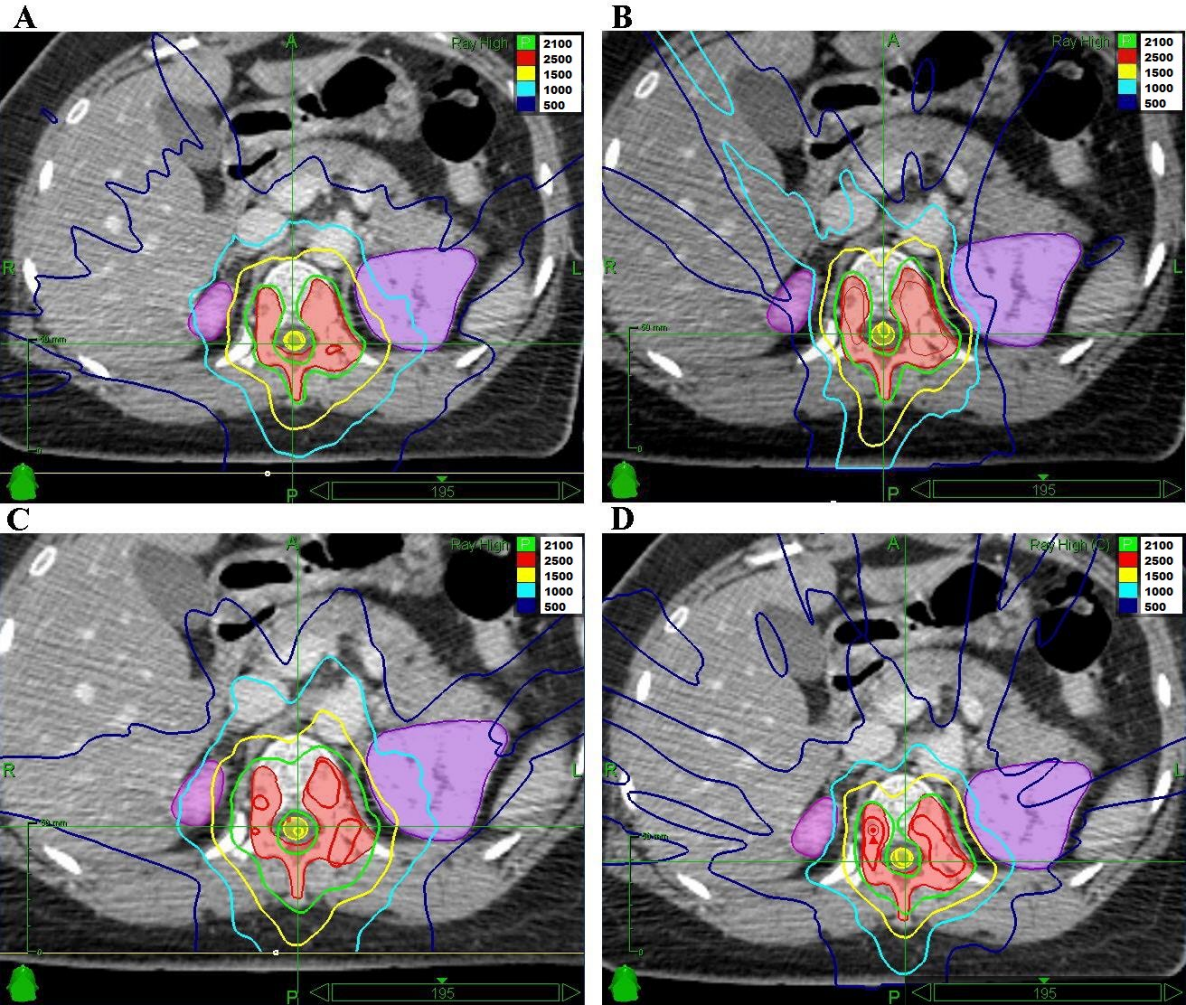
Examples of final treatment plans: (a) Case 2 with good conformity, but low coverage (clinically acceptable); (b) Case 4 with good conformity, but high spinal cord dose (clinically not acceptable); (c) Case 8 with low monitor units and treatment time, but high spinal cord dose and low conformity;(d) Case 10 with balanced trade‐off between spinal cord doses, coverage, conformity, and monitor units.

As with the treatment planning approaches, the resulting treatment plans also varied substantially among the participating CyberKnife centers (Table 2). Examples are shown in Fig. 2. Eight TP (80%) reached the minimum required coverage of 95% in the PTV while the prescription isodose levels ranged from 65%–82%, allowing maximum doses in the PTV between 25.6‐32.3 Gy. The conformity index (CI) ranged from 1.5 to 2.3 (mean 1.7), resulting mainly from optimization of the tuning and shell structures and not from the collimator selection. For the critical organs, the strict maximum spinal cord volume constraints were exceeded by three TP (30%) and the maximum kidney limit by one TP (10%). The maximum spinal cord dose ranged from 17.1‐20.7 Gy (mean 18.8 Gy) and the maximum kidney dose ranged from 10.6‐22.3 Gy (mean 18.6 Gy). The Volume10 cm $V_{10\text{Gy}}$ ranged 233‐424 cc (mean 313 cc). The number of resulting treatment beams and beam directions (nodes) ranged from 118‐232 (mean 180) and 48‐92 (mean 70), respectively. The number of nodes and the MU per node limitation did not directly correlate with the skin dose; however, the highest skin dose (19 Gy) was noted to be with the lowest number of nodes and a high MU per node allowance (case 5). The final plan MU varied between 29,507 and 74,276 which did not directly correlate with MU optimization or initial maximum MU limitation. The final estimated treatment time ranged from 39 to 89 min per fraction (mean 56), which included 5 min per fraction for patient setup and alignment.

**Table 2:** Final treatment plans of the participating centers.

	*Case*									
*Results*	*C 1*	*C 2*	*C 3*	*C 4*	*C 5*	*C 6*	*C 7*	*C 8*	*C 9*	*C 10*
Beams	118	209	226	228	187	232	161	139	171	132
Nodes	64	92	89	82	48	85	53	48	82	59
PTV Coverage (%)	96.3	91.4	95.7	95.4	95.9	95.2	95.1	92.2	95.0	96.0
Spinal Cord $V_{14\text{Gy}}$ (cc)	2.0	2.0	2.4	2.6	2.0	1.7	1.9	3.1	1.6	1.9
Spinal Cord $V_{18\text{Gy}}$ (cc)	0.0	0.0	0.1	0.2	0.0	0.0	0.1	0.3	0.1	0.0
Re‐Rx Dose (Gy)	21.8	22.0	20.8	20.3	22.5	21.9	21.1	19.0	21.4	22.2
Conformity Index	1.8	1.5	1.8	1.5	1.7	1.7	1.7	2.3	1.8	1.6
Monitor Units (MU)	34,130	38,856	62,378	74,276	56,357	43,106	42,558	29,507	43,923	38,936
Treatment Time^a^ (min)	39	52	89	72	51	65	55	44	49	42
PTV Dmin (Gy)	17.5	16.9	16.8	17.3	18.1	16.2	16.6	17.7	14.7	16.3
Spinal Cord Dmax (Gy)	18.4	17.1	18.9	20.7	18.0	18.4	18.9	19.3	19.7	18.3
Kidney Dmax (Gy)	22.3	19.4	10.6	18.3	20.4	18.9	19.6	21.9	20.4	14.2
Skin5mm Dmax (Gy)	10.0	10.0	11.0	15.0	19.0	10.0	15.0	15.0	15.0	12.0
Volume10cm $V_{10\text{Gy}}$ (cc)	330	367	238	362	352	249	265	424	313	233

a Treatment time is measured per fraction including setup and robot motion time.

PTV = planning target volume; $V_{\text{xGy}}=\text{volume receiving X Gy}$; Volume10cm = 10 cm volume surrounding the PTV; Re‐Rx Dose = Max PTV D95% (Gy) subject to NTCP limits.

### C. Review panel ranking

Similar to the variations in the treatment planning approaches and the resulting treatment plans, the reviewers varied substantially in their individual category and final plan ranking (Table 3). Four plans received a high rating (1) and also a low rating (4) by at least one reviewer and two plans each received ratings between 1‐3 and 2‐4, respectively, demonstrating the different clinical priorities of different participating reviewers. Nevertheless, the review panel did agree on case 8, which was ranked low (4) and also had the highest spinal cord doses, and on case 10, which was ranked high (1‐2) and had a balanced mix of low monitor units, spinal cord doses, and high conformity and coverage. On summing up the individual reviewer rankings and generating an overall ranking (1‐4), two plans (20%) were ranked in the excellent category (1), four plans (40%) in the average category (2), two plans (20%) in the below average category (3), and two plans in the poor category (4). The three plans that did not obey the strict spinal cord limits also received poor overall ranking (rank sum >30) and were deemed not acceptable for treatment.

**Table 3:** Reviewer ranking of the final treatment plans (1=good or excellent, 2=average, 3=below average, 4=poor).

*Human Ranking Reviewer*	*Case*									
	*C 1*	*C 2*	*C 3*	*C 4*	*C 5*	*C 6*	*C 7*	*C 8*	*C 9*	*C 10*
RV 1	1	3	4	3	2	1	2	4	1	2
RV 2	2	2	3	3	4	1	2	4	3	1
RV 3	1	1	3	4	2	1	2	4	3	2
RV 4	2	2	4	4	2	1	2	4	2	1
RV 5	2	2	3	4	2	2	2	4	4	1
RV 6	4	3	2	4	2	2	2	4	2	1
RV 7	3	3	3	3	1	1	1	4	2	1
RV 8	1	1	4	2	2	4	3	4	4	1
RV 9	1	1	3	4	2	1	1	4	3	1
RV 10	2	3	3	3	2	1	3	4	4	^1^
Min	1	1	2	2	1	1	1	4	1	1
Max	4	3	4	4	4	4	3	4	4	2
Mean	1.9	2.1	3.2	3.4	2.1	1.5	2.0	4.0	2.8	1.2
Median	2.0	2.0	3.0	3.5	2.0	1.0	2.0	4.0	3.0	1.0
SD.	1.0	0.9	0.6	0.7	0.7	1.0	0.7	0.0	1.0	0.4
Rank Sum	19	21	32	34	21	15	20	40	28	12
Final Rank	2	2	3	4	2	1	2	4	3	1

### D. Mathematical ranking

The mathematical ranking demonstrated similar results to the mean overall reviewer ranking (Table 4), which potentially enables this mathematical formula to be used for overall quality comparison of treatment plans. Nine out of 10 (90%) final mathematical rankings agreed with the overall review panel ranking. Weighting the spinal cord dosimetry by a factor of 2 did not change the ranking of the plans. A negative difference (worse mathematical than reviewer ranking) was found in case 7, which could be due to the fact this plan had no visible negative flaw (e.g., high spinal cord doses or low conformity), but the resulting indices were mostly below the average compared to the other nine plans. The mathematical ranking sum was, however, not a direct indicator for the clinical acceptability of the treatment plan, as case 7 had a higher overall rank than case 3, but had the 4th lowest overall rank in the review panel ranking.

**Table 4:** Mathematical ranking of the treatment plans ((1=good or excellent, 2=average, 3=below average, 4=poor)).

*Mathematical Ranking Index*	*Case*									
	*C 1*	*C 2*	*C 3*	*C 4*	*C 5*	*C 6*	*C 7*	*C 8*	*C 9*	*C 10*
PTV Coverage	1	4	1	2	1	2	2	4	2	1
Cord $V_{14\text{Gy}}$ ^(cc)^	2	2	3	4	2	1	2	4	1	2
Cord $V_{18\text{Gy}}$ ^(cc)^	1	1	3	4	1	1	3	4	2	1
Re‐Rx Dose	2	1	3	3	1	1	3	4	2	1
Conformity Index	3	1	3	1	2	2	3	4	3	2
Monitor Units	1	2	4	4	3	2	2	1	2	2
Treatment Time^a^	1	2	4	3	2	3	2	1	2	1
PTV Dmin	2	2	2	2	1	3	3	1	4	3
Spinal Cord Dmax	2	1	3	4	2	2	3	3	3	2
Kidney Dmax	4	3	1	2	4	3	3	4	4	1
Skin 5 mm Dmax	3	2	1	3	4	1	3	3	2	1
Volume10cm $V_{10\text{Gy}}$	3	4	1	3	3	1	1	4	2	1
Rank Sum (nonweighted)	25	25	29	35	26	22	30	37	29	18
Final Rank	2	2	3	4	2	1	3	4	3	1
Weighted Sum (factor 2 on spinal cord)	28	28	35	43	29	24	35	45	32	21
Final Weighted Rank	2	2	3	4	2	1	3	4	3	1

a Treatment time in measured per fraction including setup and robot motion time.

PTV = planning target volume; $V_{\text{xGy}}=\text{volume receiving X Gy}$; Volume10cm = 10 cm volume surrounding the PTV; Re‐Rx Dose = Max PTV D95% (Gy) subject to NTCP limits.

### E. Study agreement

Based on the results of this study, agreement was found by the study participants for the following points:

1. A well‐balanced treatment plan in terms of all plan indices is generally preferable over treatment plans with extreme quality in only a few categories (e.g., case 10 and case 6 vs. case 3 and 4 with very low surrounding dose and high conformity, but high spinal cord dose and MU as trade‐off) as long as any strict critical structure limits are not violated. Well‐balanced trade‐offs between higher and lower prioritized objectives may be made based on the treatment intent (e.g., curative, palliative, reirradiation).
2. Producing high‐quality treatment plans for complex tumor shapes may not require complex treatment planning with multiple tuning structures or lengthy optimization scripts (e.g., case 10). Due to the sequential steps implementation of SMOO in MultiPlan (version 4.5), the optimizer could be stuck on a local optimum after a few steps.^11^ The use of short and simple optimization scripts^(23)^ and/or manual adjustment of the constraints combined with iterative optimization with different scripts can help to explore the various trade‐offs of the planning constraints and goals in order to create a well‐balanced treatment plan for a specific case.
3. Regardless of the distance to the target area, critical structures should in general be dosimetrically optimized according to the as‐low‐as‐reasonably‐achievable (ALARA) principle (i.e., the minimization of critical structure maximum doses is advised) if this is not detrimental to the plan quality (e.g., case 10 where the maximum kidney dose was limited without impairing the spine dose or target coverage). Generally, it was agreed upon by the study participants that good clinical practice is the blocking of sensitive critical structures far away from the target area (e.g., blocking beams incidental to the eyes during general cranial radiosurgery).
4. The skin dose and potential hot spots outside the direct proximity of the target should be evaluated after the mandatory dose calculation in the full planning CT dose grid. During optimization in small dose grids, the skin and hot spot dose can be controlled using MU per Node constraints (e.g., 200‐350 MU per node per fraction) or larger tuning shell volumes (e.g., 3‐5 cm surrounding the PTV due to the 6 MV beam buildup).
5. Higher inhomogeneity and therefore higher maximum dose to the PTV does not automatically lead to better dose gradients or better treatment plan quality (e.g., case 4 and 7). If clinically justified, a certain degree of inhomogeneity to the target dose is unlikely to impair the plan quality, as long as the maximum‐dose regions are generally kept within the central gross tumor volume.
6. The Iris variable‐aperture collimator may not necessarily lead to better dosimetric plan quality (e.g., case 6 vs. 10 with similar dosimetry). However, the Iris collimator can reduce treatment time^(18)^ and allows easier collimator selection, potentially reducing the overall treatment planning time. Small collimator sizes, high number of beams, and high monitor units do not always and automatically lead to high dose conformity and high treatment plan quality even when treatment time is not evaluated (e.g., case 4 vs. case 10).
7. Allowing beams crossing the arms is acceptable if the arms are fully visible in the planning CT and if their repositioning during treatment is reproducible with confidence. Blocking incidental beams to the arms may reduce plan quality due to beam angle limitations (e.g., case 3 and 4).

## IV. DISCUSSION

This international benchmark study for robotic radiosurgery demonstrated various approaches to inverse treatment planning and plan quality preferences throughout a range of CyberKnife centers. Nevertheless, agreement on plan quality and basic approaches to treatment planning could be reached using an independent review panel and plan quality ranking functions. While our planning guidelines are specific for spinal robotic radiosurgery, our presented method may also be useful for providing reference information for quality improvement and quality control and enabling further investigation of homogenization and standardization of radiotherapy and radiosurgery treatment planning. While contouring guidelines have been widely accepted (e.g., for spine SBRT^(24)^) and treatment planning studies using different radiation devices or treatment delivery benchmark studies (e.g., for spine SBRT^(25)^) are common, the quality of radiotherapy treatment planning has been only rarely investigated and often only on a national level.^26^, ^27^, ^28^, ^29^, ^30^ Since treatment planning is strongly user‐dependent and quality control of treatment planning is largely lacking, this study was our first approach to provide a method for quality comparison and to define planning guidelines, in our first case for spinal robotic radiosurgery.

In the selection of the participants, as there are now more than 300 installed CyberKnife units worldwide, we tried to find a balanced mix between very experienced (>1 yrs) and rather new users (<1 yr) to the CyberKnife. However, the experience of the user did not reflect in the resulting plan quality — for example, the planner of case 6 (2nd in ranking) did not have access to MultiPlan version 4.5 before this study. From the results of our study, it also became clear that treatment plans generated with simple optimization scripts (i.e., case 6 or case 10) may yield better results than those with complex scripts and multiple tuning structures. Since a very simple treatment planning technique yielded good results, our planning guidelines may also be valid for simple targets and other indications, requiring further validation. Practical guidelines for robotic radiosurgery treatment planning can be found in Appendix A.

A simple approach to CyberKnife treatment planning could be to start with known critical structure limitations as hard constraints,^(21)^ maximize the target coverage in the first step and minimize the dose to the tuning shell structures for dose conformity (e.g., planning for case 5 and 6). This simple approach can already lead to high‐quality treatment plans.^(23)^ Furthermore, the resulting shell doses could be used as constraints in a second optimization script in order to minimize the dose to the critical structures (e.g., planning for case 2). It may be noted that some specific optimization steps (e.g., minimum volume dose optimization) could potentially yield inferior results compared to others especially with nearby critical structures (i.e., planning for cases 3, 4, and 8 with the highest spinal cord doses). To still archive acceptable results while using the minimum volume dose constraint, the goal relaxation value needs to be large enough to warrant the competition between the different objectives in those cases. Another approach to CyberKnife treatment planning could be to manually derive the tuning shell and critical structure doses by subsequently reducing the maximum and volume constraints until the target receives ≤95 coverage (e.g., planning of case 10). This iterative optimization technique is the basis of the sequential multiobjective optimization.^(11)^ Yet, since the implementation of SMOO in MultiPlan (since version 3.0), plan optimization scripts are used more commonly.

Regardless of the type of optimization, skin entry doses and hot spots outside the target should be controlled as to avoid severe skin or organ reactions. This is not an easy task with the CyberKnife due to the many beam directions and the generally small optimization grids used during planning. A dose calculation covering all beam entry spots is strongly recommended, and actions to limit the skin and hot spot dose during optimization are proposed in our planning guidelines. Furthermore, it is well known that different treatment planning protocols can lead to large differences in organ‐at‐risk sparing,^31^, ^32^ and our study also highlighted those differences when comparing the kidney maximum doses. In contrast to other studies,^(32)^ we advise that treatment planning should be generally based on the ALARA (as‐low‐as‐reasonably‐achievable) principle rather than on implementing stricter constraints for critical structures. Sensitive critical structures farther from the target may as well be blocked by beam intersection altogether to ensure the ALARA principle and to avoid multiple replanning steps.

The reviewer ranking of the plans revealed that well‐balanced treatment plans were preferred for treatment over plans with extreme quality in only a few categories. It should be noted that a plan exceeding the spinal cord constraints (i.e., case 3, 4, and 8) would have not been accepted for treatment. These plans were, therefore, rated low in Category A (main clinical objectives) and Category E (clinical assessment), providing a certain weighting in the favor of low spinal cord doses. Indeed, the plans exceeding the spinal cord limit received the lowest mean overall ranking.

Because a large review panel quality assessment is not always available in general routine practice, we also wished to provide a mathematical measure for plan quality assessment. This approach to determine the best treatment plan has been investigated,^23^, ^33^, ^34^, ^35^, ^36^ but does not yet encompass the multidimensional complexity of radiosurgery plan quality and the preferences of the treating clinicians. Our simple mathematical ranking system generated the same category as the expert review panel in 9 out of 10 cases. The inclusion of multiple indices for the spinal cord ($V_{14\text{Gy}}$, $V_{18\text{Gy}}$, $D_{\text{Max}}$) also provided a similar weighting compared to the review panel ranking in the favor of low spinal cord doses.

Nevertheless, caution is advised when using this formula, as severe treatment flaws (e.g., unacceptably high spinal cord doses in case 3) may be masked by high scores in other indices.

Penalties or a weighting factor (Table 4) on certain aspects of the treatment plan based on clinical preferences may make this simple formula more robust and adaptable to other entities and a larger number of participants, both of which are subjects for further investigation.

Limitations of our study are the number of cases (n=1) and the number of participants (n=10) and reviewers (n=10), and we are aware that both the guidelines and the ranking system would need to be validated in larger cases series to be applicable for spine or robotic radiosurgery in general. Nevertheless, this is the first study and attempt at standardization of treatment plan and planning quality for robotic radiosurgery. We selected a single complex‐shaped tumor surrounding a critical structure to demonstrate that, even for such a challenging case, simple planning methods can lead to high‐quality treatment plans, allowing the planning guidelines to be potentially applicable to simple cases, as well. One may also argue that the whole spine could have been included in the PTV^(37)^ and the dose chosen for this treatment may be low for spine SBRT,^19^, ^20^ but as noted earlier, the patient had received preirradiation to this area limiting the spinal cord dose significantly. We believe that across the 10 treatment plans, the range of treatment plan qualities for this case was reasonably covered. Further incorporation of 17 more treatment plans into the mathematical ranking, all created by independent planner without knowledge of the results of this study, did not alter the ranking significantly nor did it change any point of the planning guidelines. However, we cannot state with absolute certainty that the treatment planning approach or the quality of the best‐ranked treatment plan in this study (i.e., case 10) is the best achievable for this patient. Further improvements to robotic radiosurgery optimization of spine SBRT have already been demonstrated^38^, ^39^, ^40^ and the new InCise (Accuray Inc.) multileaf collimator for the CyberKnife may further improve plan quality. ^(41)^ Nevertheless, our study demonstrates that a best practice approach to CyberKnife treatment planning is feasible, and further cases and a measure for treatment planning quality improvement are under investigation.

## V. CONCLUSIONS

This multi‐institutional study illustrates different inverse treatment planning approaches and treatment preferences for spinal robotic radiosurgery. Despite their wide variation in experience, training, and clinical preferences, the participants', reviewers', and the mathematical formula's agreement on the preferable treatment plan quality and on the inverse treatment planning techniques indicates that agreement on treatment planning and plan quality can be reached for spinal robotic radiosurgery. The provided data and method for benchmarking and the planning guidelines could potentially improve the consistency of treatment planning for robotic radiosurgery in the future.

## ACKNOWLEDGMENTS

The authors would like to thank Etienne Lessard (Accuray) for his support in this study and Mikail Gezginci (Accuray) for his helpful remarks. Centre Oscar Lambret has a collaboration agreement with Accuray. Jimm Grimm has a patent for the DVH Evaluator issued. Otherwise, the authors declare that they have no competing interests.

## COPYRIGHT

This work is licensed under a Creative Commons Attribution 4.0 International License.

## Supporting information

Supplementary Material

Supplementary Material

Supplementary Material

## Appendix A: Methods for Creating Treatment Plans Using the Sequential Multi‐Objective Optimization (SMOO) Feature

In this supplement material we are presenting simple methods for creating treatment plans for robotic radiosurgery with the CyberKnife, using the Sequential Multi‐Objective Optimization (SMOO) feature based on the results of our international benchmark study, the experiences by the participants, and published literature. Please note that these approaches to treatment planning may not be optimal for every target to be treated with the CyberKnife, but they may provide a quick and good first solution for further optimization and fine‐tuning. Please also consider that there may be other planning approaches leading to similar results, and we do not claim that treatment planning has to be performed as presented in this supplemental material to create high quality CyberKnife treatment plans.

## TREATMENT PLANNING FOR ROBOTIC RADIOSURGERY

### A. Shell structures

Shell structures are used to control the dose conformity, dose falloff, and the skin entry doses, as well as hot spots outside the target area depending on their distance away from the target. A general rule for the creation of shell structures is that, the smaller the target and the smaller the collimators, the closer the shell structures can be and the less shell structures are needed. Furthermore, for intracranial targets, generally closer shell structures are used than for extracranial target. We therefore provide a range of sizes which need to be adjusted according to the size of the target. Be advised that not all distances for shell structures should be used for optimization as they may be limiting the optimization of the planning target volume (PTV) dose or of steep dose falloffs towards organs at risk (OAR).

#### A.1 Optimization of high dose conformity

A shell structure of 1‐3 mm may be used to limit higher doses outside the PTV, especially if the PTV has a nonspherical shape. Please note that extreme limiting or optimizing this shell structure may lead to significant trade‐offs with respect to PTV coverage or OAR doses.

#### A.2 Optimization of prescription dose conformity and dose falloff

The use of two shell structures with a distance between each other of 5‐10 mm is recommended for the optimization of the prescription dose conformity (${\text{Shell}}_{\text{Rx}}$) and the proximate dose falloff (${\text{Shell}}_{\text{DFO}}$). For intracranial targets, 3‐7 mm for the ${\text{Shell}}_{\text{Rx}}$ and 10‐15 mm for the ${\text{Shell}}_{\text{DFO}}$ and, for extracranial targets, 5‐10 mm for the ${\text{Shell}}_{\text{Rx}}$ and 15‐20 mm for the ${\text{Shell}}_{\text{DFO}}$ are generally useful shell distances.

#### A.3 Optimization of low dose conformity and beam entry doses

Due to the 6 MV beam buildup, a shell structure of 30‐50 mm, depending on target and collimator size, may be used to limit and optimize skin doses and hot spots outside the proximate target region. Such a shell structure can be helpful for multiple targets to avoid hot spots due to beam intersections.

### B. Plan setup

The dose calculation or optimization grid should be set such that the outer shell structures and any directly relevant OAR are within the grid. The optimization grid resolution in MultiPlan version 4.x is variable, with a maximal number of possible constraint points of either 64×64×64 (low resolution), 128×128×128 (medium resolution), or based on the number of CT voxel (high resolution). It has to be kept in mind that the actual location of the constraint points are based on the used resolution and may vary between the different resolutions. Furthermore, it may be noted that the constraint points may be located not at the direct boundary of the different volumes, leading to differences in optimized and displayed dose values. Please also consider that the use of large shell structures or OARs further away from the target (i.e, everything furtherthan 20 mm from the PTV) may in general require the use of higher dose grid resolution during optimization and hence slow down the planning time significantly. A commissioned density model with tissue inhomogeneity correction should be used, and contour correction should be selected which corrects for false beam depth calculation for beams not entering at convex tissues. As a good clinical practice procedure, critical OARs (e.g., eyes, optic nerves, spinal cord, esophagus, testicles, and many others) not included in the dose calculation grid should be blocked to plan according to the ALARA (as‐low‐as‐reasonably‐achievable) principle if the plan quality is elsewise not compromised. Arms may not need to be blocked if repositioning is guaranteed during treatment. Finally, any critical patient attachments (e.g., pacemakers) and patient cutoffs on the CT may be blocked as the dose calculation may be incorrect if beams enter those regions.

### C. Optimization setup

If no larger shell structure is used, maximum monitor units (MU) per node may be limited (e.g., 200‐350 MU per node per fraction) to avoid skin entry doses and hot spots outside the target region. As the MU per beam limitation does not add a significant value to the plan quality (given the correct use of shell structures), it could be omitted; however, it can additionally be used to limit hot spots outside the PTV. As the MU per beam limitation also does not significantly reduce the optimization results or treatment time (given the correct use of time and beam reduction), a limitation of 50%–75% of the allowed MU per node can be selected for maximum MU per beam. The total MU may be limited to avoid long treatment times and the use of too many small collimated beams, potentially resulting in cold spots inside the PTV. A general rule of thumb is 1000‐1500 MU per Gy for single intracranial and 1500‐2000 MU per Gy for single extracranial complex‐shaped targets. If a high number of MUs and small beams cannot be avoided, an additional PTV boost structure (e.g., PTV minus 3‐5 mm) may be used during optimization to avoid low doses in the center of the PTV.

### D. Organs at risk

Critical OAR within the optimization grid should be limited using maximum and pseudo‐volume‐constraints, according to common and internal guidelines and published dose limitations^(21)^ and according to the ALARA principle. Please note that the volume constraints in SMOO are not strict volume constraints, but rather voxel constraints (including a slack factor) for the subvolume closest to the PTV. As a result, manual adjustments for the pseudo‐volume‐constraints in volume or dose may be necessary to ensure the planning system does not violate the actual volume dose limit. Furthermore, if a low optimization grid resolution is used, OAR dose constraints may in general need manual adjustments (i.e., be decreased), since the constraint points may not necessarily be located at the OAR boundary. Regarding the ALARA principle, critical OAR within the optimization grid may also be blocked if clinically justified, however, the plan quality may be significantly reduced if they are close to the PTV. Regardless of maximum dose or volume limitations, critical OAR should always be dosimetrically optimized based on the maximum achievable dose falloff from the PTV. As a general rule for the CyberKnife, the maximum dose falloff can be in the order of 3‐4 Gy per mm, which could be used to judge the achieved dose to very close or very radiation‐sensitive critical organs. On the other hand, a minimum dose falloff in any direction of 1‐2 Gy per mm should generally be achievable, which could be used as a general guideline to optimize OAR further away from the PTV, according to the ALARA principle.

### E. Collimator selection

The selection of suited collimators for any given PTV shape is nontrivial. Studies have demonstrated that the use of multiple collimators is beneficial to the plan quality, but that more than three collimators may not be beneficial — even worsening the plan quality as the number of initial generated beams per collimator will be reduced.^(18)^ In detail, for three collimators, 3000 initial beams are used (1000 beams per collimator), whereas for 12 collimators, only 6000 initial beams are used (500 beams per collimator) due to limitations in CPU memory and optimization time. For initial collimator selection for nonisocentric treatments, generally smaller collimators are preferred for intracranial targets (e.g., 50%–75% of the tumor diameter) and larger collimators are preferred for extracranial and especially moving targets (e.g., 75%–90% of the tumor diameter). When using multiple collimators, they should generally be well distributed over tumor diameter ranges (e.g., using 50%, 70%, and 90% of the tumor diameter as opposed to use 50%, 55%, and 60% of the tumor diameter). Furthermore, the approximate beam's eye view target dimensions should be taken into account for collimator selection. Please consider that using small collimators may not necessarily result in better dose conformity or dose falloff, especially in extracranial targets. An example for this point is the use of the 5 mm collimator for spinal lesion where studies have demonstrated that the use of the 7.5 mm collimator achieved a better dose falloff towards the spinal cord, even without using any MU limitations.^(18)^ For spinal cases similar to the one presented in our benchmark study, the use of a small (7.5‐10 mm), a medium (12.5‐20 mm), and a large (25‐30 mm) collimator led to the best overall results. Similar results were found for prostate treatment, where the combination of a small (10‐15 mm), a medium (20‐35 mm), and a large (40‐50 mm) collimator yielded the best optimization results.^(18)^ Be advised that determining those three collimators may require multiple iterations, and treatment planning time may be shortened by selecting a higher number of collimators (e.g., four to six), however, pointing out the possibility again that plan quality could be reduced with a larger number of collimators.

### F. First optimization

The first optimization should be as simple as possible (e.g., 2‐3 steps, low resolution) and can be used to determine the maximum shell doses as they are dependent on collimator selection, MU limitations, and target shape and generally not known *a priori.* Furthermore, the first optimization may be used to determine a good collimator selection and should be quick to reduce the overall planning time. Therefore, the number of constraint points should be limited to lower than 10,000 for the PTV and lower than 5,000 for OAR or shell structures. The optimization grid resolution and number of constraint points may then be increased in the subsequent optimizations to generate the final plan. A simple script to explore basic plan qualities is presented in the following steps:

Step 1: Optimize PTV Coverage (OCO at Rx+X Gy with Y Gy relaxation). Please note that using minimum PTV volume dose limits (i.e., using Optimize PTV Minimum Dose OMI or Optimizing PTV Homogeneity OHI) may limit the optimization result in the subsequent steps and should be avoided if clinically justified. Dose‐volume optimization (DVL) in the first step may also be used, but be aware that the optimization will take longer in that case. Furthermore please note that, if a low optimization grid resolution is used, the optimization target dose may need manual adjustment (i.e., be increase) since the constraint points may not necessarily be located at the PTV boundary.
Step 2: Optimize ${\text{Shell}}_{\text{Rx}}$ Conformity (OCI at 0 Gy with Z Gy relaxation). Consider that the minimal ${\text{Shell}}_{\text{Rx}}$ maximum dose is generally unknown *a priori.* Hence, no useful maximum constraint can be set for any shell structure used in the optimization steps. Please be advised that using a specific optimization dose target for OCI other than 0 Gy violates the ALARA principle. The same is also true for optimizing maximum OAR doses (OMA).
Step 3: Optimize ${\text{Shell}}_{50}$ Conformity (OCI at 0 Gy). Due to the sequential nature of SMOO, the priority of clinical objectives is reflected in the order of optimization steps. If prescription isodose conformity is the higher objective (e.g., for intracranial targets), the ${\text{Shell}}_{\text{Rx}}$ may be optimized in Step 2. If lower isodose conformity is the higher objective (e.g., for moving extracranial targets), the ${\text{Shell}}_{50}$ may be optimized in Step 2. Please consider that further steps can be used to optimize other shell structures, OAR maximum doses, or total MUs; however, the longer the optimization script, the less priority the specific objective will receive, which may result in minimal to no improvement in plan quality after a few steps.

Relaxation factors after a specific step control the flexibility the optimizer has in the subsequent step. Hence, they control the trade‐off between two steps and, therefore, between two specific objectives. A low relaxation factor (e.g., 0‐10 cGy) will likely result in no‐to‐minimal improvement for the next objective, whereas a higher relaxation factor (e.g., 75‐100 cGy) will likely result in a significant improvement for the next objective. Pausing after each step to determine the trade‐off that one is willing to make for the next step or running multiple iterations of the same script with different relaxation factors may help to determine a good set of relaxation factors in order to create a well‐balanced treatment plan.

### G. Second optimization

For many targets without any close critical structures (e.g., peripheral brain or lung tumors), the first optimization script alone (e.g., with higher resolution) may be used to create high‐quality treatment plans.^(23)^ If there are close critical structures, either a manual adjustment of maximum or pseudo‐volume‐constraints or a second optimization script can be used to optimize OAR doses, which requires the setting of the maximum dose constraints for the shell structures determined in the first optimization. A simple script for OAR optimization is presented in the following steps:

Step 1: Optimize PTV Coverage (OCO at Rx+X Gy with Y Gy relaxation) ‐ see First Optimization above.
Step 2: Optimize OAR Mean Dose (OME at H Gy with Z Gy relaxation). Using a specific optimization dose target for OME may likely result in a lower volume which this OAR receives at the given dose target compared to optimizing the overall OAR mean dose (OME at 0 Gy). Please be advised that the use of relaxation factors in this step may result in a higher maximum dose in the OAR compared to the defined maximum constraint (i.e., by Z Gy) due to the implementation of the relaxation factors in SMOO. If other OARs require further optimization, those OARs will be prioritized according to their position in the optimization script and their relaxation factors. If similar OARs have the same priority (e.g., optic nerves for a target with the same distance to left and right optic nerve), a sum contour may be used for optimization.
Step 3: Optimize Total Monitor Units (OMU). Please note that the optimization of MU does not automatically lead to shorter treatment times, as number of used beams and nodes could increase during this optimization.

### H. Fine‐tuning

In many cases fine‐tuning (i.e., the manual adjustment of constraints and relaxation factors) can result in an overall improvement of plan quality due to the stepwise implementation of the optimization in SMOO. Fine‐tuning may also be used to explicitly minimize critical structure maximum doses and total MU to plan, according to the ALARA principle.

### I. Time, node, and beam reduction

After a satisfying optimization, the number of beams is generally high and the resulting beam set contains a higher number of small weighted beams. It has been agreed upon that beams with lower than 10 MU per fraction should not be used for treatment due to nonlinearity in dose deposition. Also, the number of beams, and with that, the total treatment time can generally be significantly reduced by more than 25% without losing significant plan quality. Time reduction is a feature in SMOO where low‐weighted beams and beam directions (nodes) are removed from the optimization problem and new beams are introduced which are geometrically fitted to the target in the beam's eye view before optimizing the plan with the set optimization script. The plan quality may actually improve by using time reduction; however, too many time reduction iterations may significantly reduce the plan quality due to the removal of too many nodes.

Node and beam reduction, on the other hand, will only remove low‐weighted nodes and beams and reoptimize with the remaining nodes and beams. For node and beam reduction, fine‐tuning of the optimization script may be advised in order to keep the plan quality (e.g., increasing maximum shell doses by 5‐25 cGy, increasing MU per node by 5‐25, or tightening of relaxation factors to maintain PTV coverage). Please be advised that time, node, and beam reduction may still result in a beam set with small weighted beams due to the linear programming in the optimization of SMOO. If a minimum MU constraint would be added (i.e., all beams have to have 0 or higher than X MU), the result would be a mixed integer optimization exponentially increasing the optimization time. Therefore, multiple iterations of time, node, and beam reduction or final removal of small weighted beams may be necessary.

### J. Multiple targets and simultaneous integrated boost

Multiple targets and simultaneous integrated boosts (SIB) may also not require a more complex planning approach than a single target; however, it may require the use of a sum (multiple target) of differential (PTV minus boost) contours for the generation of common shell structures and for simultaneous optimization of all targets in the planning script (given they should receive the same prescription dose). For multiple targets with different prescription doses or for SIB, a prioritization of a specific PTV or of PTV versus Boost may be difficult (i.e., which structure to optimize first) and it may be helpful to optimize each structure alone to determine the achievable shell doses or necessary MU for that structure before optimizing multiple structures in a single script. The use of larger relaxation factors and potential switches in priority during time, node, and beam reduction may be further helpful to optimize multiple targets and SIB with different prescription doses. Regardless of dose, great care is advised to avoid dose bridges in between multiple targets.

### K. Final plan evaluation

Final plan evaluation should always be made on a dose calculation, which includes all beam entry and exit spots, to evaluate OAR previously outside the dose optimization grid, dose fingers (dose >30% of the maximum dose) and skin entry and exit doses. Beam entry points, which could be outside the visible CT skin structure, should be validated before prescribing the dose to the PTV. No agreement was reached regarding PTV dose homogeneity, coverage, and conformity. Common practice is the prescription to the 60%–80% isodose (excluding prostate treatments), with 98%–100% coverage for intracranial and 95%‐98% coverage for extracranial targets. The conformity index for a noncomplex‐shaped target should generally be lower than 1.2.
