# Supplementary material for: Inverse treatment planning for spinal robotic radiosurgery: an international multi‐institutional benchmark trial
Source: J Appl Clin Med Phys. 2016 May 8;17(3):313–30. doi: 10.1120/jacmp.v17i3.6151 (PMC5690905; doi:10.1120/jacmp.v17i3.6151)
Supplement: Supplementary file 2 — Supplementary Material [file ACM2-17-313-s002.docx]

**Supplement Material**

In this supplement material we are presenting simple methods for creating treatment plans for robotic radiosurgery with the CyberKnife (Accuray Inc, CA, USA) using the Sequential Multi-Objective Optimization (SMOO) feature based on the results of our international Bench Mark study and experiences by the participants and published literature. Please note that these approaches to treatment planning may not be optimal for every target to be treated with the CyberKnife, but they may provide a quick and good first solution for further optimization and fine tuning. Please also consider that there may be other planning approaches leading to similar results and we do not claim that treatment planning has to be performed as presented in this supplement material to create high quality CyberKnife treatment plans.

**Treatment Planning for Robotic Radiosurgery**

*Shell Structures*

Shell structures are used to control the dose conformity, dose fall-off and the skin entry doses as well as hot spots outside the target area depending on their distance away from the target. A general rule for the creation of shell structures is that the smaller the target and the smaller the collimators the closer the shell structures can be and the less shell structures are needed. Furthermore, for intracranial targets generally closer shell structures are used than for extracranial target. We therefore provide a range of sizes which need to be adjusted according to the size of the target. Be advised that not all distances for shell structures should be used for optimization as they may be limiting the optimization of the planning target volume (PTV) dose or of steep dose fall-offs towards organs at risk (OAR).

- *Optimization of high dose conformity:* A shell structure of 1-3mm may be used to limit higher doses outside the PTV especially if the PTV has a non-spherical shape. Please note that extreme limiting or optimizing this shell structure may lead to significant trade-offs with respect to PTV coverage or OAR doses.
- *Optimization of prescription dose conformity and dose fall-off:* The use of two shell structures with a distance between each other of 5-10mm is recommended for the optimization of the prescription dose conformity (Shell_Rx_) and the proximate dose-fall off (Shell_DFO_). For intracranial targets 3-7mm for the Shell_Rx_ and 10-15mm for the Shell_DFO_ and for extracranial targets 5-10mm for the Shell_Rx_ and 15-20mm for the Shell_DFO_ are generally useful shell distances.
- *Optimization of low dose conformity and beam entry doses:* Due to the 6 MV beam build-up a shell structure of 30-50mm depending on target and collimator size may be used to limit and optimize skin doses and hot spots outside the proximate target region. Such a shell structure can be helpful for multiple targets to avoid hot spots due to beam intersections.

*Plan Setup*

The dose calculation or optimization grid should be set such that the outer shell structures and any directly relevant OAR are within the grid. The optimization grid resolution in MultiPlan version 4.x is variable with a maximal number of possible constraint points of either 64 x 64 x 64 (low resolution), 128 x 128 x 128 (medium resolution) or based on the number of CT voxel (high resolution). It has to be kept in mind that the actual location of the constraint points are based on the used resolution and may vary between the different resolutions. Furthermore, it may be noted that the constraint points may be located not at the direct boundary of the different volumes leading to differences in optimized and displayed dose values. Please also consider that the use of large shell structures or OAR further away from the target (i.e, everything further away than 20mm from the PTV) may in general require the use of higher dose grid resolution during optimization and hence slow down the planning time significantly. A commissioned density model with tissue inhomogeneity correction should be used and contour correction should be selected which corrects for false beam depth calculation for beams not entering at convex tissues. As a good clinical practice procedure, critical OAR (e.g., eyes, optic nerves, spinal cord, esophagus, testicles, and many more) not included in the dose calculation grid should be blocked to plan according to the ALARA (As Low As Reasonably Achievable) principle if the plan quality is elsewise not compromised. Arms may not need to be blocked if repositioning is guaranteed during treatment. Finally, any critical patient attachments (e.g., pacemakers) and patient cut-offs on the CT may be blocked as the dose calculation may be incorrect if beams entering those regions.

*Optimization Setup*

If no larger shell structure is used, maximum monitor units (MU) per node may be limited (e.g., 200-350 MU per Node per fraction) to avoid skin entry doses and hot spots outside the target region. As the MU per beam limitation does not add a significant value to the plan quality (given the correct use of shell structures) it could be omitted, however, it can additionally be used to limit hot spots outside the PTV. As the MU per beam limitation also does not significantly reduce the optimization results or treatment time (given the correct use of time and beam reduction) a limitation of 50-75% of the allowed MU per node can be selected for maximum MU per beam. The total MU may be limited to avoid long treatment times and the use of too many small collimated beams potentially resulting in cold spots inside the PTV. A general rule of thumb is 1000-1500 MU per Gy for single intracranial and 1500-2000 MU per Gy for single extracranial complex shaped targets. If a high number of MU and small beams cannot be avoided an additional PTV boost structure (e.g., PTV minus 3-5mm) may be used during optimization to avoid low doses in the center of the PTV.

*Organs At Risk*

Critical OAR within the optimization grid should be limited using maximum and pseudo volume constraints according to common and internal guidelines and published dose limitations [21] and according to the ALARA principle. Please note that the volume constraints in SMOO are not strict volume constraints, but rather voxel constraints (including a slack factor) for the sub-volume closest to the PTV. Due to that, manual adjustments for the pseudo volume constraints in volume or dose may be necessary to ensure the planning system does not violate the actual volume dose limit. Furthermore, if a low optimization grid resolution is used, OAR dose constraints may in general need manual adjustments (i.e., be decreased) since the constraint points may not necessarily be located at the OAR boundary. Regarding the ALARA principle, critical OAR within the optimization grid may also be blocked if clinically justified, however, the plan quality may be significantly reduced if they are close to the PTV. Regardless of maximum dose or volume limitations, critical OAR should always be dosimetrically optimized based on the maximum achievable dose fall-off from the PTV. As a general rule for the CyberKnife, the maximum dose fall-off can be in the order of 3-4Gy per mm which could be used to judge the achieved dose to very close or very radiation sensitive critical organs. On the other hand, a minimum dose fall-off in any direction of 1-2Gy per mm should generally be achievable, which could be used as a general guideline to optimize OAR further away from the PTV according to the ALARA principle.

*Collimator Selection*

The selection of suited collimators for any given PTV shape is non-trivial. Studies have demonstrated that the use of multiple collimators is beneficial to the plan quality, but that more than 3 collimators may not be beneficial even worsening the plan quality as the number of initial generated beams per collimator will be reduced [18]. In detail, for 3 collimators 3000 initial beams are used (1000 beams per collimator) whereas for 12 collimators only 6000 initial beams are used (500 beams per collimator) due to limitations in CPU memory and optimization time. For initial collimator selection for non-isocentric treatments generally smaller collimators are preferred for intracranial targets (e.g., 50-75% of the tumor diameter) and larger collimators are preferred for extracranial and especially moving targets (e.g., 75-90% of the tumor diameter). When using multiple collimators they should generally be well distributed over tumor diameter ranges (e.g., using 50%, 70% and 90% of the tumor diameter as opposed to use 50%, 55% and 60% of the tumor diameter). Furthermore, the approximate beam eye view target dimensions should be taken into account for collimator selection. Please consider that using small collimators may not necessarily result in better dose conformity or dose fall-off especially in extracranial targets. An example for this point is the use of the 5mm collimator for spinal lesion where studies have demonstrated that the use of the 7.5mm collimator achieved a better dose fall-off towards the spinal cord even without using any MU limitations [18]. For spinal cases similar to the one presented in this Bench Mark study the use of a small (7.5-10mm), a medium (12.5-20mm) and a large (25-30mm) collimator lead to the best overall results. Similar results were found for prostate treatment where the combination of a small (10-15mm), a medium (20-35mm) and a large (40-50mm) collimator yielded the best optimization results [18]. Be advised that determining those 3 collimators may require multiple iterations and treatment planning time may be shortened by selecting a higher number of collimators (e.g., 4-6), however, pointing out the possibility again that plan quality could be reduced with a larger number of collimators.

*First Optimization*

The first optimization should be as simple as possible (e.g., 2-3 steps, low resolution) and can be used to determine the maximum shell doses as they are dependent on collimator selection, MU limitations and target shape and generally not known *a priori*. Furthermore, the first optimization may be used to determine a good collimator selection and should be quick to reduce the overall planning time. Therefore, the number of constraint points should be limited to lower than 10,000 for the PTV and lower than 5,000 for OAR or shell structures. The optimization grid resolution and number of constraint points may then be increased in the subsequent optimizations to generate the final plan. A simple script to explore basic plan qualities is presented in the following bullet points:

- *Step 1: Optimize PTV Coverage (OCO at Rx + X Gy with Y Gy Relaxation):* Please note that using minimum PTV volume dose limits (i.e., using Optimize PTV Minimum Dose OMI or Optimizing PTV Homogeneity OHI) may limit the optimization result in the subsequent steps and should be avoided if clinically justified. Dose volume optimization (DVL) in the first step may also be used, but be aware that the optimization will take longer in that case. Furthermore please note, that if a low optimization grid resolution is used the optimization target dose may need manual adjustment (i.e., increase) since the constraint points may not necessarily be located at the PTV boundary.
- *Step 2: Optimize Shell_Rx_ Conformity (OCI at 0 Gy with Z Gy Relaxation):* Consider that the minimal Shell_Rx_ maximum dose is generally unknown *a priori*. Hence, no useful maximum constraint can be set for any shell structure used in the optimization steps. Please be advised that using a specific optimization dose target for OCI other than 0Gy violates the ALARA principle. The same is also true for optimizing maximum OAR doses (OMA).
- *Step 3: Optimize Shell_50_ Conformity (OCI at 0 Gy):* Due to the sequential nature of SMOO the priority of clinical objectives is reflected in the order of optimization steps. If prescription isodose conformity is the higher objective (e.g., for intracranial targets) the Shell_Rx_ may be optimized in step 2. If lower isodose conformity is the higher objective (e.g., for moving extracranial targets) the Shell_50_ may be optimized in step 2. Please consider that further steps can be used to optimize other shell structures, OAR maximum doses or total MU, however, the longer the optimization script the less priority the specific objective will receive which may result in minimal to no improvement in plan quality after a few steps.
- *Relaxation factors:* Relaxation factors after a specific step control the flexibility the optimizer has in the subsequent step. Hence, they control the trade-off between two steps and therefore between two specific objectives. A low relaxation factor (e.g., 0-10cGy) will likely result in no to minimal improvement for the next objective whereas a higher relaxation factor (e.g., 75-100cGy) will likely result in a significant improvement for the next objective. Pausing after each step to determine the trade-off willing to make for the next step or running multiple iterations of the same script with different relaxation factors may help to determine a good set of relaxation factors in order to create a well-balanced treatment plan.

*Second Optimization*

For many targets without any close critical structures (e.g., peripheral brain or lung tumors) the first optimization script (e.g., with higher resolution) may be solely used to create high quality treatment plans [23]. If there are close critical structures either a manual adjustment of maximum or pseudo volume constraints or a second optimization script can be used to optimize OAR doses which requires the setting of the maximum dose constraints for the shell structures determined in the first optimization. A simple script for OAR optimization is presented in the following bullet points:

- *Step 1: Optimize PTV Coverage (OCO at Rx + X Gy with Y Gy Relaxation):* See First Optimization
- *Step 2: Optimize OAR Mean Dose (OME at H Gy with Z Gy Relaxation):* Using a specific optimization dose target for OME may likely result in a lower volume which this OAR receives at the given dose target compared to optimizing the overall OAR mean dose (OME at 0Gy). Please be advised that the use of relaxation factors in this step may result in a higher maximum dose in the OAR compared to the defined maximum constraint (i.e., by Z Gy) due to the implementation of the relaxation factors in SMOO. If other OAR require further optimization those OAR will be prioritized according to their position in the optimization script and their relaxation factors. If similar OAR have the same priority (e.g., optic nerves for a target with the same distance to left and right optic nerve) a sum contour may be used for optimization.
- *Step 3: Optimize Total Monitor Units (OMU):* Please note that the optimization of MU does not automatically lead to shorter treatment times as number of used beams and nodes could increase during this optimization.

*Fine Tuning*

In many cases fine tuning (i.e., the manual adjustment of constraints and relaxation factors) can result in an overall improvement of plan quality due to the step-wise implementation of the optimization in SMOO. Fine tuning may also be used to explicitly minimize critical structure maximum doses and total MU to plan according to the ALARA principle.

*Time, Node and Beam Reduction*

After a satisfying optimization, the number of beams is generally high and the resulting beam set contains a higher number of small weighted beams. It has been agreed upon that beams with lower than 10 MU per fraction should not be used for treatment due to non-linearity in dose deposition. Also, the number of beams and with that the total treatment time can generally be significantly reduced by more than 25% without losing significant plan quality. Time reduction is a feature in SMOO where low weighted beams and beam directions (nodes) are removed from the optimization problem and new beams are introduced which are geometrically fitted to the target in the beams-eye-view before optimizing the plan with the set optimization script. The plan quality may actually improve by using time reduction; however, too many time reduction iterations may significantly reduce the plan quality due to the removal of too many nodes.

Node and beam reduction on the other hand, will only remove low weighted nodes and beams and re-optimize with the remaining nodes and beams. For node and beam reduction, fine tuning of the optimization script may be advised in order to keep the plan quality (e.g., increasing maximum shell doses by 5-25cGy, increasing MU per node by 5-25 or tightening of relaxation factors to maintain PTV coverage). Please be advised that time, node and beam reduction may still result in a beam set with small weighted beams due to the linear programming in the optimization of SMOO. If a minimum MU constraint would be added (i.e., all beams have to have 0 or higher than X MU) the result would be a mixed integer optimization exponentially increasing the optimization time. Therefore, multiple iterations of time, node and beam reduction or final removal of small weighted beams may be necessary.

*Multiple Targets and Simultaneous Integrated Boost*

Multiple targets and simultaneous integrated boosts (SIB) may also not require a more complex planning approach than a single target; however, it may require the use of a sum (multiple target) of differential (PTV minus boost) contours for the generation of common shell structures and for simultaneous optimization of all targets in the planning script (given they should receive the same prescription dose). For multiple targets with different prescription doses or for SIB a prioritization of a specific PTV or of PTV versus Boost may be difficult (i.e., which structure to optimize first) and it may be helpful to optimize each structure alone to determine the achievable shell doses or necessary MU for that structure before optimizing multiple structures in a single script. The use of larger relaxation factors and potential switches in priority during time, node and beam reduction may be further helpful to optimize multiple targets and SIB with different prescription doses. Regardless of dose, great care is advised to avoid dose bridges in between multiple targets.

*Final plan evaluation*

Final plan evaluation should always be made on a dose calculation which includes all beam entry and exit spots to evaluate OAR previously outside the dose optimization grid, dose fingers (dose > 30% of the maximum dose) and skin entry and exit doses. Beam entry points, which could be outside the visible CT skin structure, should be validated before prescribing the dose to the PTV. No agreement was reached regarding PTV dose homogeneity, coverage and conformity. Common practice is the prescription to the 60-80% isodose (excluding prostate treatments) with 98-100% coverage for intracranial and 95-98% coverage for extracranial targets. The conformity index for a non-complex shaped target should generally be lower than 1.2.
